# Research Progress in Steam Explosion for Biomass Pretreatment and Its Application to Pyrolysis and Gasification

**DOI:** 10.3390/molecules31071158

**Published:** 2026-03-31

**Authors:** Guanya Liu, Lifeng Wang, Wenhao Lian, Zhongling Zhang, Xiaogang Hao, Jiansheng Zhang

**Affiliations:** 1 College of Chemical Engineering and Technology, Taiyuan University of Technology, Taiyuan 030024, China; 2 Shanxi Pharmaceutical Vocational College, Taiyuan 030031, China; 3 Shanxi Province Key Laboratory of Chemical Process Intensification, North University of China, Taiyuan 030051, China; 4 Shanxi Research Institute for Clean Energy, Tsinghua University, Taiyuan 030032, China

**Keywords:** biomass, energy conversion, steam explosion, pyrolysis, gasification

## Abstract

Steam Explosion (SE) is a relatively newly developed physicochemical pretreatment method that has received increasing attention since it can effectively upgrade biomass for further utilization. During SE, biomass is first exposed to high-temperature, high-pressure steam and then rapidly depressurized. This process efficiently breaks down the lignocellulosic structure, reduces moisture content, and increases fixed carbon and calorific value. It also enhances biomass grindability and densification, making it more suitable as a renewable solid fuel. This review carefully discusses the fundamental principles of SE and its effects on particle characteristics. Then, the types of SE reactors (mainly composed of batch reactors and continuous reactors) are systematically compared, and the challenges in scaling up and commercialization are discussed. Also, the characteristics of pyrolysis or gasification of biomass pretreated by SE are described in detail. Studies indicate that SE is beneficial for the enhancement of product quality. Finally, the prospects and future challenges in the development of SE (including superheated steam explosion, reaction kinetics improvement, and heat and mass transfer intensification) are presented and discussed.

## 1. Introduction

The increasing demand for clean energy has driven the advancement of biomass-based conversion technologies, establishing them as a key approach for the sustainable utilization of renewable resources [1,2]. The primary method for bioenergy conversion involves transforming the chemical energy naturally stored in biomass into accessible and affordable energy forms—such as heat, electricity, liquid biofuels, or syngas—through thermochemical, biochemical, or physicochemical processes [3]. Numerous energy conversion technologies have been developed, including pyrolysis, gasification, anaerobic digestion, and fermentation [2,4]. However, the inherent recalcitrance and variable composition of biomass present significant challenges to efficient conversion, often requiring substantial energy input and posing considerable technical and economic barriers. Therefore, pretreatment is considered one of the most promising methods for converting bioenergy from lignocellulosic biomass [5].

Thus, pretreatment has emerged as a critical upstream process in biomass energy conversion, as it can effectively overcome the inherent recalcitrance of lignocellulosic feedstocks, enhance conversion efficiency, and unlock the full energy potential of biomass resources [6]. Researchers have proposed various pretreatment methods, including physical methods (e.g., grinding and milling), physicochemical methods (e.g., hydrothermal carbonization and steam explosion), chemical methods (e.g., alkali leaching, dilute acid leaching, and organic solvent pretreatment), and biological methods (e.g., fermentation) [7,8,9]. Numerous methods have been developed for the efficient processing of lignocellulosic biomass. Among these, physical methods consume less energy and are relatively simple; however, they are generally ineffective at breaking down the lignin and hemicellulose structures of biomass [10]. Chemical methods typically require acids or alkalis under mild reaction conditions, but the resulting biomass fuel contains higher levels of harmful ions, which can cause equipment corrosion and increase the difficulty of wastewater treatment challenges [7]. Biological methods are low-cost and operate at ambient temperatures, yet their conversion rates are too slow to meet industrial demands [11]. Therefore, although the aforementioned methods play important roles in their respective fields, they are not well-suited for large-scale energy conversion of biomass resources. Physicochemical methods supply heat to biomass, inducing both physical and chemical changes that enable efficient and large-scale pretreatment. These methods combine the advantages of the other approaches and have become a research focus for biomass energy conversion [12,13,14].

Steam explosion (SE), which involves high-temperature cooking and rapid decompression explosions, is a well-known physicochemical biomass pretreatment method. It combines physical and chemical effects to deliver significant benefits such as environmental friendliness, cost savings, and high processing efficiency, thereby promoting the efficient conversion of lignocellulosic biomass [15]. In SE, biomass is directly heated by the latent heat released from higher temperature steam (180–240 °C), in contrast to hydrothermal carbonization, which involves longer heating times (30–240 min) and natural cooling [16]. During the process, autohydrolysis occurs within 5–15 min, followed by instant depressurization. A combination of high-temperature cooking and sudden depressurization changes the biomass structure, including density, particle size, porosity, grindability, and hydrophobicity. By enhancing the fuel properties of untreated biomass, these structural changes make it more suitable for converting biomass into fuel.

This review critically examines the features of various reactor types, including batch and continuous designs, while offering a thorough overview of the basic idea and research advancements in SE. In contrast to earlier reviews that mainly focused on biochemical conversion routes, this work methodically assesses the effects of SE on the physicochemical characteristics of biomass and its performance in thermochemical conversion processes, specifically pyrolysis and gasification. A detailed summary of the gasification and pyrolysis characteristics of treated biomass is presented to clarify how pretreatment affects product distribution and conversion efficiency. The review also discusses future directions, such as heat/mass transfer intensification and superheated steam explosion (SHSE), as well as the integration of SE with leaching for ash mitigation. This review attempts to direct future research and commercialization efforts in biomass energy conversion by bridging the gap between basic mechanisms and industrial scalability.

## 2. Biomass SE Pretreatment

### 2.1. Overview of Biomass SE Process

Biomass SE involves two main processes: high-temperature cooking and rapid decompression explosion stages. During the high-temperature cooking stage, biomass is exposed to high-temperature (180–240 °C) and high-pressure (1–3.5 MPa) steam [17,18]. Hemicellulose is hydrolyzed by acetic acid released from acetyl groups, while lignin undergoes depolymerization, and the strength of cellulose is reduced. A subsequent rapid depressurization to atmospheric pressure breaks the rigid fiber structure, converting the biomass into a fibrous, dispersed solid. The sudden pressure release generates shear forces that break glycosidic bonds and hydrogen bonds, thereby facilitating enzymatic hydrolysis of cellulose and hemicellulose into monomeric hexoses and pentoses [19]. As a result, SE significantly changes the architecture of biomass cell walls and yields a slurry consisting of a water-soluble fraction rich in hemicellulose-derived sugars and a water-insoluble fraction enriched in cellulose and lignin [6]. After SE pretreatment, the solid yield typically ranges from 60% to 90%. This yield is influenced by biomass type, severity factors (temperature and residence time), and the treatment of post-treatment washing or fractionation [15,17]. This yield range reflects the trade-off between hemicellulose removal and the preservation of cellulose and lignin for downstream applications. Understanding and optimizing this balance is key to tailoring SE for specific end-uses, whether for solid fuel upgrading, biochemical production, or thermochemical conversion. It is worth noting that acid or alkaline is introduced in some cases to leach the biomass before SE to improve the process effectiveness. However, the ions introduced in the leaching process will cause various problems in the following SE process, such as boiler corrosion and wastewater problems. The adverse effects may even persist during later gasification, combustion, or other energy conversion processes [20,21].

The key parameters of SE include temperature, pressure, and residence time, which directly influence the quality of biomass fuel treated by SE. In addition to operation parameters, the properties of the biomass also affect the production quality of SE pretreatment, such as moisture content and size. Considering that temperature and residence time are independent operating variables, Chornet [22] proposed the severity factor kinetic model ***R_0_*** (where ***R_0_ = t* *×* *exp^[(T-100)/14.75]^***) to evaluate the intensity of SE pretreatment. This enables the determination and understanding of how operating parameters affect biomass, thereby facilitating the modeling, optimization, and scale-up of the SE pretreatment. Apart from the severity factor kinetic model, many scholars have proposed SE energy consumption models [23,24,25].

**Figure 1:**
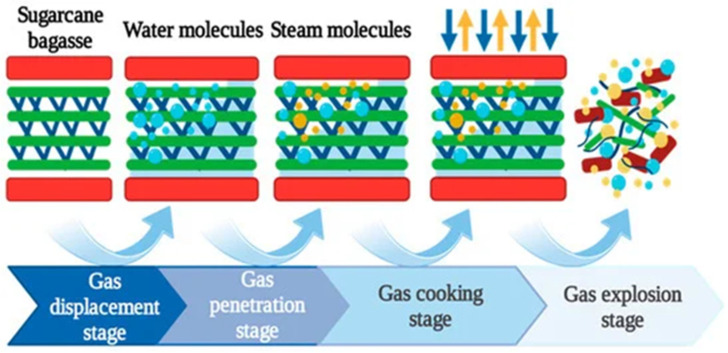
A schematic representation of the biomass SE process, depicting the sequential stages of gas-phase displacement, penetration, cooking, and explosion. Reprinted from Ref. [28].

Based on the principles of heat and mass transfer between steam and biomass, Sui [26,27] innovatively divided the SE process into four typical stages according to changes in temperature (pressure) and moisture content, namely: gas-phase displacement, gas-phase penetration, gas-phase cooking, and gas-phase explosion stages (Figure 1). The gas-phase displacement stage refers to the phase in which dry biomass is restored to an optimal moisture state through a rehydration process prior to steam explosion treatment. During this stage, water enters the pores of the material, displacing and expelling the original air. Effective rehydration is believed to significantly enhance the steam explosion effect.

However, the increase in internal moisture content also affects the subsequent heat transfer method and efficiency, thereby indirectly influencing energy consumption. In the gas-phase permeation stage, high-pressure steam rapidly penetrates into the material. A large amount of steam is consumed to heat the low-temperature material, which is closely related to maintaining temperature (pressure). The heat transfer effectiveness and uniformity of the steam during this stage directly impact both the steam explosion outcome and energy consumption. Once the temperature (pressure) inside the vessel reaches the set value, the process enters the gas-phase cooking stage. The material is maintained under high-temperature and high-pressure conditions for a specific duration, during which a series of thermochemical reactions occur. After the holding time reaches the preset value, the process proceeds to the gas-phase explosion stage, where the material is rapidly discharged under instantaneous pressure release, producing the explosive effect. Additionally, a key evaluation indicator for SE energy consumption was proposed—the mass of steam consumed by per unit mass of dry materials (where $m^{\prime }=\frac{Q_{t}/\left(h_{ge}-h_{f0}\right)}{m}$) [26]. These studies found that SE operation strategies, such as reducing the moisture content of the biomass and appropriately crushing the biomass to increase bulk density, can effectively lower energy consumption, enhance heat transfer efficiency, and improve the economic viability of the SE pretreatment [28].

SE is a versatile technology applicable to a wide range of lignocellulosic feedstocks. However, the response of each feedstock to the treatment can vary significantly due to differences in their chemical composition and physical structure. For example, hardwoods and agricultural residues are generally easier to pretreat with SE than softwoods [29]. This is attributed to the lower lignin content in hardwoods, which is more readily depolymerized than the highly condensed guaiacyl (G) lignin found in softwoods [30]. The physical characteristics of the feedstock, such as initial particle size and moisture content, also play a crucial role in ensuring uniform steam penetration and heating, thereby affecting the homogeneity of the production.

The treatment of the condensate produced from high-pressure steam in the SE process merits closer examination. This liquid fraction, being similar to prehydrolysate, is a complex stream comprising monomeric and oligomeric sugars (primarily xylose, glucose, and arabinose), acetic acid, furfural, and other degradation products [31]. Constituting 10–30% of the original biomass weight, this underutilized resource presents a substantial opportunity for biofuel and biochemical synthesis. Its valorization potential is demonstrated by its efficacy in anaerobic digestion for methane production, with yields comparable to conventional biomass [32]. Furthermore, the recovered sugars are amenable to fermentation for ethanol production or serve as carbon sources for microbial oil accumulation. Consequently, adopting an integrated biorefinery framework that facilitates the concurrent valorization of both solid and liquid streams is crucial for enhancing the overall economic viability and environmental performance of the SE process.

### 2.2. Effects of SE on the Particle Property

During the high-temperature cooking stage, the hemicellulose in biomass is extensively degraded through autohydrolysis reactions, leading to a decrease in the O/C and H/C ratios and an increase in the calorific value of the biomass [31,33,34,35,36], which makes the fuel characteristics closer to those of coal [37,38]. Detailed comparison of the proximate analysis, ultimate analysis, and calorific value data of the untreated and treated biomass is shown in Table 1. From Table 1, the changes in the ultimate analysis and proximate analysis of agricultural (flax straw, wheat straw, hybrid *pennisetum*, and reed) and forestry waste (EFB) are largely similar. However, the ash content exhibits different patterns of change depending on the pretreatment conditions and equipment used. High-quality biomass fuel should have very low ash content; therefore, it is necessary to investigate methods to reduce the ash content of SE products.

**Figure 2:**
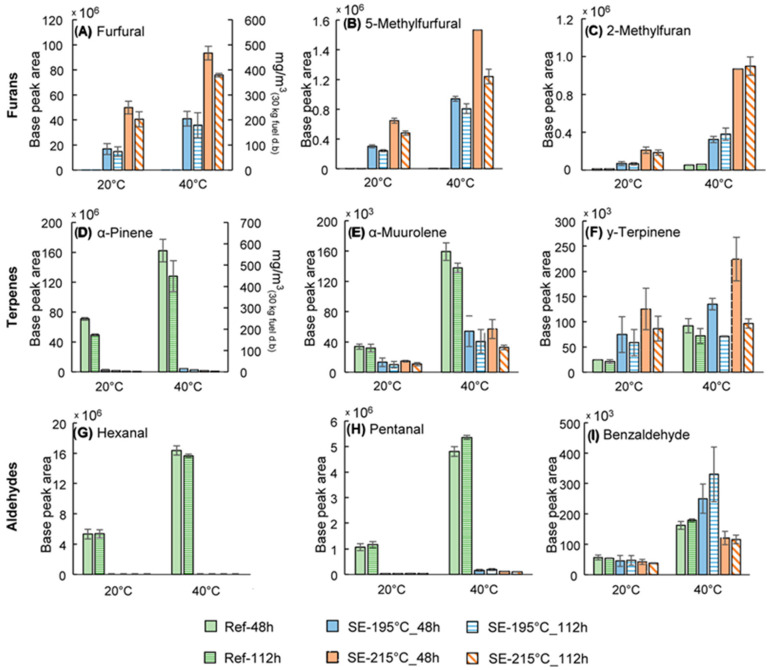
Comparison of emissions of various compounds from wood storage at different SE temperatures.(Comparison of furans emissions: (**A**) furfural, (**B**) 5-methylfurfural, and (**C**) 2-methylfuran; terpenes: (**D**) α-pinene, (**E**) α-murrolene, and (**F**) γ-terpinene; aldehydes: (**G**) hexanal, (**H**) pentanal, and (**I**) benzaldehyde, in the off-gas from reference and steam exploded (at 195 and 215 °C) wood chips at 20 or 40 °C storage temperatures, after 48 h and 112 h storage, using base peak areas for the respective compound. For furfural and α-pinene, the concentrations are also presented in mg/m^3^.) Reprinted with permission from Ref. [39]. Copyright 2026 American Chemical Society.

The emission of Volatile organic compounds (VOCs) throughout the biomass fuel lifecycle, including production, transportation, and storage, poses a critical safety risk due to potential fire hazards and health impacts. This issue should not be overlooked. Eleonora [39] conducted VOCs release experiments using a mixture of Scots pine and Norway spruce wood chips. From Figure 2, it can be found that SE pretreatment can reduce the emissions of certain VOCs known to have health impacts, such as hexanal. However, this process also resulted in the generation of other potentially more hazardous compounds, including furfural, 5-methylfurfural, and 2-methylfuran. Therefore, it is essential to closely monitor and eliminate the content of furfural during the SE process and subsequent processing stages, such as pelletization.

**Table 1:** Proximate analysis, ultimate analysis, and calorific value data of the biomass treated by SE.

Feedstock	SE Condition	Proximate Analysis (wt%, Dry Basis)		Ultimate Analysis (wt%)						
		Volatile Matter (%)	Ash (%)	Fixed Carbon (%)	C (%)	H (%)	O (%)	N (%)	S (%)	HHV (MJ/Kg)
flax straw [40]	Untreated	82.5	3.6	13.9	47.1	5.9	43.1	0.1	0.1	19.7
	215 °C/1 min	78.8	3.4	17.8	49.3	6.2	40.3	0.8	0.1	20.4
wheat straw [31]	Untreated	77.13	5.43	17.44	44.06	5.77	44.46	0.42	0.08	17.47
	220 °C/5 min	73.39	5.4	21.21	48.65	5.66	39.93	0.47	-	19.4
spruce bark	Untreated	75.41	2.99	21.6	49.1	5.74	41.9	0.33	0.06	19.5
	220 °C/5 min	68.62	3.77	27.61	54.34	5.42	36.43	0.33	-	21.51
EFB	Untreated	70.86	8.31	20.83	44	5.38	41.49	1.02	0.06	17.23
	220 °C/5 min	68.49	9.31	22.2	48.22	5.34	36.61	1.12	-	19.14
hybrid *Pennisetum* [23]	Untreated	73.52	2.8	23.68	44.75	4.65	49.79	0.38	0.43	17.66
	200 °C/10 min	71.38	3.23	25.39	46.74	3.69	48.96	0.28	0.33	18.15
	225 °C/10 min	67.49	3.23	29.28	50.14	4.84	44.43	0.24	0.35	19.56
	250 °C/10 min	64.01	3.94	32.05	52.16	3.62	43.67	0.26	0.29	19.79
	275 °C/10 min	33.96	6.94	59.1	66.23	3.42	29.62	0.44	0.29	24.83
Reed [41]	Untreated	78.93	5.32	15.75	44.21	5.85	44.15	0.4	0.08	17.65
	300 °C, 2.5 MPa, 60 min	67.62	5.94	26.44	51.19	5.43	36.93	0.41	0.1	20.33
	330 °C, 2.5 MPa, 60 min	63.59	6.62	29.79	52.38	5.31	35.15	0.43	0.11	20.77
	360 °C, 2.5 MPa, 60 min	61.06	8.28	30.66	53.7	5.41	31.06	0.46	0.09	21.63

C (%): The carbon content of biomass. H (%): The hydrogen content of biomass. O (%): The oxygen content of biomass. N (%): The nitrogen content of biomass. S (%): The sulfur content of biomass. HHV (MJ/Kg): The higher heating value of biomass.

After SE, biomass typically undergoes a pelletization process to further enhance its density, mechanical properties, and hydrophobicity. SE has an obvious effect on the characteristics of pellets since the biomass particle size and lignin structure will be significantly changed [42,43]. During the steaming cooking stage, high-temperature steam activates the lignin in the biomass, causing its structure to rearrange and transition from a glassy state to a molten state [44,45]. This forms new chemical bonds within the lignin-cellulose chains, enhancing hydrophobicity and particle strength, and allowing the lignin to act as a binder in subsequent pellet processes [34]. In the following explosion stage, the liquid water, which has penetrated among the biomass fibers under high temperature and high pressure, suddenly vaporizes, causing rapid expansion in volume and destroying the biomass structure, increasing porosity, and reducing particle size [46]. After SE pretreatment, as shown in Table 2, the biomass fuel treated by pelletization shows a significant increase in Meyer hardness and maximum breaking strength, a slight decrease in asymptotic modulus, and improved hydrophobicity, indicating a slight reduction in material rigidity and a potential increase in toughness. These characteristics exhibit outstanding fuel performance. However, it should be noted that both compression energy and extrusion energy generally increase after the SE treatment, suggesting that treated biomass requires more energy for pelletization.

**Table 2:** Mechanical properties and energy input for pelletization of treated and untreated EFB, PKS and DF [46].

Biomass		Meyer Hardness (N/mm^2^)	Maximum Breaking Strength (MPa)	Asymptotic Modulus (GPa)	Compression Energy (J/g)	Extrusion Energy (J/g)	Moisture Content (%)
EFB	Untreated	1.82	57.71	0.72	30.15	0.99	13.5
	220 °C, 5 min	2.97	94.06	0.51	44.50	5.81	7.3
PKS	Untreated	1.35	42.70	0.62	32.61	3.95	13.5
	220 °C, 5 min	1.95	61.83	0.59	31.81	5.16	6.7
DF	untreated	1.60	18.00	1.33	20.45	0.49	6.9
	220 °C, 5 min	6.60	59.30	1.19	36.25	1.17	6.0

### 2.3. The Techno-Economic Analysis of SE

Biomass fuel pellets constitute a class of compressed lignocellulosic solid biofuels derived from feedstocks including sawdust, agricultural straw, and forestry byproducts. These fuels exhibit high volumetric and energetic density, enhanced storability via compact form, efficient combustion kinetics, and reduced carbon footprint compared to traditional solid fuels [6]. They are extensively adopted as coal alternatives in industrial heating systems, thermoelectric power generation, and boiler applications. Recent growth of the pellet industry in North America, Europe, and Asia corresponds to global renewable energy mandates and carbon mitigation objectives, with Canada and the United States emerging as leading producers and exporter nations. Large-scale production and deployment; however, encounter operational constraints: first, Seasonal and geographic variability in biomass availability causes significant cost volatility, particularly during collection, logistics, and storage of agronomic residues [18]; second, Standard pellets demonstrate compromised hydrophobic properties and reduced calorific content, impairing combustion efficiency and long-term storage integrity; third, While torrefaction and SE pretreatments enhance pellet quality through process optimization, they necessitate substantial capital expenditures and operational investments, with economic feasibility remaining suboptimal. The energetic input and ecological impacts of pellet fabrication processes also require rigorous life-cycle assessment.

SE pretreatment functions as a moderate-capital-input biomass densification technology, bridging the cost-performance spectrum between conventional pelletization and torrefaction-based approaches. As illustrated in Table 3, analysis of sawdust and oat straw feedstocks reveals equipment expenditures for SE scenarios ranging from US$2.13 million to US$2.68 million, with cumulative capital requirements of US$15.86 million to US$19.96 million [47]. This represents a 70% reduction relative to torrefaction systems, which require US$54.49 million to US$60.34 million in investment. Operational expenditure distribution indicates feedstock procurement (25–43%), site-specific infrastructure (21–36%), and labor (16–27%) as principal cost categories, with feedstock price volatility serving as the critical economic driver. Market viability calculations suggest SE pellet selling prices of US$200.5/t (sawdust) and US$208.4/t (oat straw), surpassing conventional alternatives (US$113.4–118.7/t) yet remaining 28–30% below torrefied products (US$283.4–298.7/t). Financial performance metrics demonstrate internal rates of return of 8–10%, payback periods of 6.27–6.78 years, and project sustainability under stable feedstock availability and cost-competitive biomass procurement.

**Table 3:** Comparison of techno-economic analysis for conventional pelletization, steam explosion, and torrefaction [47].

Cost Category	Conventional Pelletization	Steam Explosion	Torrefaction
Equipment Purchase Cost (million USD)	1.27–1.32	2.13–2.68	7.33–7.86
Total Capital Investment (million USD)	9.50–9.92	15.86–19.96	54.49–60.34
Minimum Selling Price (USD/t)	113.4–118.7	200.5–208.4	283.4–298.7
Internal Rate of Return (IRR)	11–19%	8–10%	Not profitable
Payback Period (years)	2.48–2.59	6.27–6.78	4.07–4.64

## 3. The Equipment of SE Pretreatment

Multiple types of reactors are employed in SE pretreatment, which can be categorized into batch, semi-continuous, and continuous reactors based on their operation mode. Among these, batch and continuous reactors are the most commonly used types of SE equipment.

### 3.1. Batch Reactors of SE

**Figure 3:**
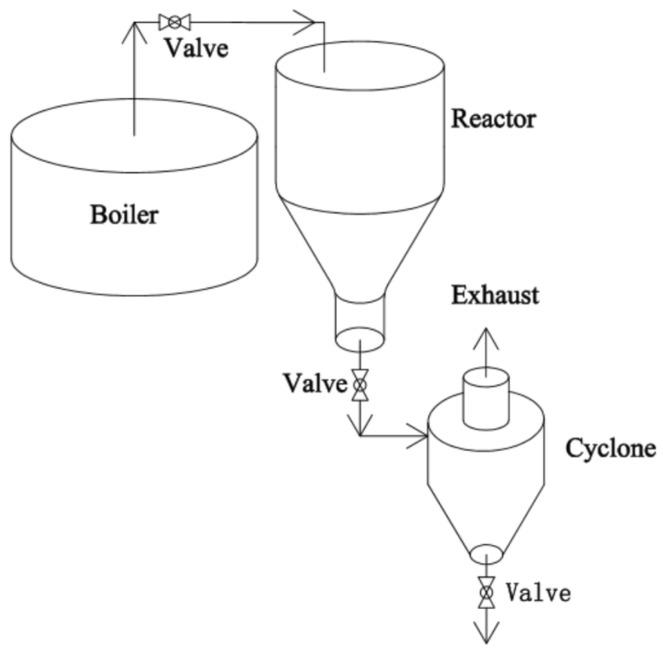
Schematic diagram of batch reactors of SE.

Batch reactors mainly consist of a steam generator, a cooking chamber and a receiving chamber, with a quick-opening valve at the bottom of the cooking chamber connecting to the receiving chamber (Figure 3) [18,48]. The most distinctive feature of this technology is its ability to achieve rapid pressure relief within seconds through a device called valve blow mode. During the cooking stage, the material is fed into the reactor from the top. Subsequently, after confirming the airtightness of the equipment, saturated steam is introduced and maintained for a preset duration. During this process, the latent heat released by the condensation of steam is utilized to heat the biomass. During the pressure relief process, the rapid expansion of high-pressure steam exposed to atmospheric pressure generates shear forces on the biomass structure, thereby achieving biomass size reduction. While valve blow mode is relatively simple to implement, the depressurization time of the reactor is closely related to the volume of the cooking chamber. If the volume of the cooking chamber reaches tens of cubic meters, the depressurization process may take tens of seconds, thereby diminishing the effect of the explosion. Additionally, valve blow mode presents several challenges in engineering applications: inconsistent biochemical properties, difficulty in heat recovery, susceptibility to clogging in front of the valve during explosion, and the need for additional cooling steps for the biomass treated by SE [49].

**Figure 4:**
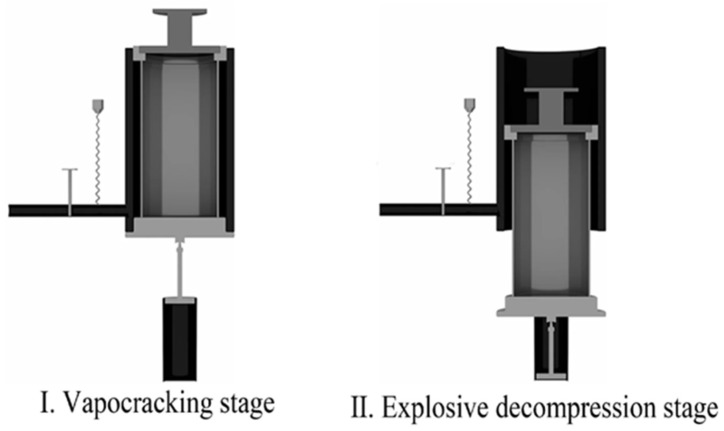
Working principal diagram of the ICSE equipment for I. vapocraking stage and II. Explosive decompression stage. Reprinted from Ref. [53].

These problems above hinder the industrial application of batch reactors. To overcome these problems, Yu [50] designed the catapult explosion mode called instant catapult steam explosion (ICSE), which consists of a high-pressure cylinder and a sliding cover that can move vertically (Figure 4). The sliding cover seals the cylinder when moved to one end and exposes it to the atmosphere when moved to the other end, enabling rapid pressure relief. Compared to traditional SE reactors (with a depressurization time of 10.785 s), the depressurization time of ICSE (0.0875 s) is significantly reduced, leading to a substantial shortening of the pretreatment cycle and notable reductions in both cost and energy consumption. The explosion power density (EPD) of the ICSE reactor can reach 35.2–48.2 MWm^−3^, which is significantly higher than the traditional valve blow mode (0–0.07 MWm^−3^), demonstrating superior fragmentation effectiveness [51]. In addition to ICSE, some scholars have also designed a pilot-operated pneumatic (POP) mode for SE, reducing the decompression time to 0.01 s, which significantly increases the porosity of the biomass [52].

Compared to continuous reactors, batch reactors (such as liquid hot water and steam explosion reactors) offer advantages including a simple structure, ease of operation, and straightforward scale-up, leading to their widespread use in laboratory and pilot-scale research. However, due to limitations in operational mode and low heat and mass transfer efficiency, batch reactors face challenges when directly applied to large-scale biomass energy conversion. These challenges primarily manifest as high heat consumption, difficulty in heat recovery, and uneven heating of the material during processing. To enhance the engineering applicability of batch reactors, future novel batch reactors should meet the following key requirements: first, Employ an optimized steam injection structure to enhance convective heat transfer between the steam and biomass, ensuring uniform heating of the stacked material and thereby improving pretreatment effectiveness; second, Minimize the explosion time as much as possible, utilizing the mechanical effects of rapid pressure release to reduce material particle size, thus lowering the energy consumption of subsequent crushing processes; and third, Implement heat recovery from the discharged waste steam, achieving energy conservation through heat integration.

### 3.2. Continuous Reactors of SE

**Figure 5:**
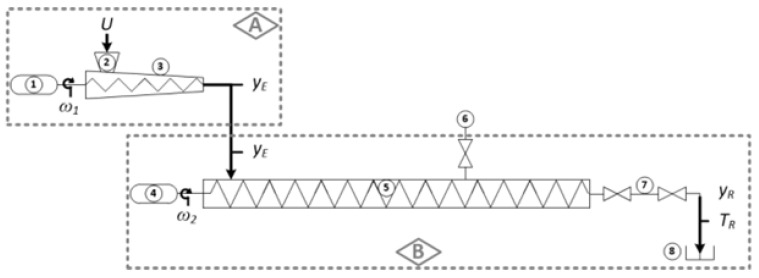
Structure diagram of the continuous reactors of SE. Reprinted with permission from Ref. [58]. Copyright 2026 American Chemical Society.

Continuous reactors typically are often achieved using a screw conveyor reactor (SCR) to achieve continuous operation—a technology that has been widely used in industries such as mining, solids handling, pulp and paper industries (eg. Hydrothermal pilot plant, Confab Industrial S/A, São Caetano do Sul, SP, Brazil) [54,55]. As shown in Figure 5, The CTR is composed of Area A (i.e., the extruder) and Area B (i.e., the CTR body), which support three process phases: extrusion (①, ②, ③), autohydrolysis (④, ⑤, ⑥), and steam explosion (⑦, ⑧). The circled numbers indicate the various parts within each area (see Table 4). After a certain residence time, it is discharged into the atmosphere through a valve blow mode [32]. Throughout this process, the amount of biomass being processed in the reactor remains essentially constant. The advantages of continuous reactors have made SE a leading pretreatment method for converting agricultural and forestry waste into bioethanol and methane, and this method has been applied on a commercial scale [56]. Pérez [57] conducted SE pretreatment on four types of biomass feedstocks (agave bagasse, corn stover, sugarcane bagasse, and wheat straw) in a continuous reactor with a processing capacity of 200 kg/day, under conditions of 180 °C and 20 min. The study found that the SE pretreatment increased the crystallinity and energy density of the biomass by removing xylan, thereby offering a promising alternative for producing advanced biofuels. Beyond its applications in the agricultural sector, continuous reactors are gradually expanding into the energy field. Valmet has established the world’s first renewable black pellet plant in Malaysia. This plant utilizes Valmet’s advanced SE system “BioTrac”, which is capable of converting 15t per hour of high-alkali salt biomass agricultural waste into homogeneous biomass fuel as a substitute for coal.

Compared to batch reactors, continuous reactors (especially screw conveyor reactors, SCR) demonstrate unparalleled advantages in the scale-up and commercial application of biomass hydrothermal pretreatment, such as high processing capacity, high thermal efficiency, strong process adaptability, and industrially validated scale-up potential [59,60]. However, this technology still faces numerous engineering challenges, including severe wear on high-pressure feeding system valves, high equipment investment costs, significant steam heat loss during operation, and high sensitivity to feedstock characteristics, leading to unstable feeding and uneven residence time distribution. To overcome these bottlenecks and promote the widespread application of continuous reactors in biomass energy conversion, future research and development should focus on the following three core directions: first, developing new materials resistant to high temperatures, high pressures, and acidic corrosion to enhance equipment lifespan and operational reliability; second, integrating efficient steam recovery and heat exchange technologies to reduce energy consumption and improve process economics; and third, conducting systematic thermal analysis, static analysis, and flow field simulation of the screw conveyor to optimize its structural design and operating parameters, thereby improving material flow characteristics and ensuring reaction efficiency and product selectivity.

## 4. Impacts of SE on Biomass Pyrolysis

Biomass pyrolysis refers to the process in which biomass is subjected to thermal energy under anaerobic conditions or with a limited air supply, leading to the breaking of chemical bonds within its macromolecular structure and the conversion of these macromolecules into smaller molecules [61,62]. As a thermochemical conversion technology, biomass pyrolysis can yield high-value products such as biochar and bio-oil, and also serves as the initial stage for the processes like gasification, combustion, and liquefaction [63]. Since SE significantly changes the structure and content of hemicellulose, cellulose, and lignin in the raw material, the pyrolysis characteristics of treated biomass particles, as well as the composition and properties of the resulting pyrolysis products, will exhibit notable differences [64].

### 4.1. Biomass Pyrolysis Characteristics

Biomass mainly consists of three components—hemicellulose, cellulose, and lignin—each exhibiting distinct pyrolysis mechanisms. Hemicellulose, mainly composed of heterogeneous polysaccharides represented by xylan, has a more intricate and branched architecture. It undergoes pyrolysis at a comparatively low temperature range of 220–315 °C. The primary pyrolysis products include liquid compounds such as water, methanol, formic acid, acetic acid, ketones, furans, and furfural, along with gaseous compounds dominated by H_2_, CO, CO_2_, and CH_4_ [65,66]. Cellulose is structured as a homogeneous linear polymer interconnected by β-1,4-glycosidic bonds. Its pyrolysis occurs predominantly within 315–400 °C, yielding products such as anhydrous saccharides, furan derivatives, carboxylic acids, and low-molecular-weight compounds [67,68]. Lignin is a three-dimensional amorphous polymer built from three phenylpropanoid units (H, G, and S) linked via C–O (β-O-4, α-O-4, 4-O-5) and C–C (5-5′, β-1, β-5′) bonds [69]. It exhibits a broad pyrolysis range from 150 °C to 700 °C [66]. During pyrolysis, lignin decomposes into phenolic monomers and dimers (from cleavage of structural units), aromatic hydrocarbons, and notably high yields of char and non-condensable gases.

The pyrolysis characteristics of biomass are commonly investigated using thermogravimetric analysis (TGA) to study its decomposition behavior and kinetic parameters [70]. Biswas [33] subjected willow wood chips to SE pretreatment and investigated their pyrolysis behavior. The study revealed that, at a pyrolysis conversion rate of 10%, the pyrolysis rates of all samples showed no significant variation. However, when the conversion rate increased to 50%, distinct differences in pyrolysis rates emerged, particularly in samples pretreated at 220 °C and 228 °C, where the pyrolysis rates were notably higher. At a pretreatment temperature of 205 °C, the reaction intensity of the sample exhibited no significant change. However, when the temperature was increased to 220 °C and 228 °C, a marked increase in reaction intensity was observed. Gu [71] applied both differential and integral kinetic analysis methods to calculate the apparent activation energy of sawdust pyrolysis. The experimental results showed that the activation energy of treated biomass was slightly lower than that of untreated samples (untreated poplar sawdust: 138.7 kJ/mol and 141.3 kJ/mol; treated poplar sawdust: 134.8 kJ/mol and 137.5 kJ/mol), indicating that SE enhanced the pyrolysis reactivity of poplar sawdust. A similar phenomenon was observed in SE experiments on straw-based biomass, where SE reduced the activation energy of corn stover by 24.13–32.56% while increasing the pre-exponential factor by 8–10% [72]. Collectively, these studies demonstrate that SE, as an effective pretreatment method, can lower the energy barrier required for biomass pyrolysis, highlighting its promising potential in the field of biomass thermochemical conversion.

### 4.2. The Characteristics of Bio-Oil Treated by SE

Bio-oil is a dark brown liquid mixture obtained by condensing the volatiles produced from the pyrolysis of biomass under anoxic or limited-oxygen conditions. It is primarily composed of oxygenated organic compounds such as alcohols, phenols, acids, and ketones, characterized by high oxygen content, acidity, and high viscosity, which can be used in energy, fuel, and chemical industries [73,74].

After SE pretreatment, the lignin structure of biomass is disrupted, generally leading to a slight increase in phenolic compounds in the pyrolysis oil [75]. Due to the inhibited decomposition of hemicellulose and cellulose, the proportion of aromatic compounds in the oil is relatively raised [76]. Wang [77,78] found that after SE pretreatment of pinewood, sweetgum, switchgrass, and corn stover, the yield of pyrolysis oil decreased, along with reduced viscosity, increased water content, a decline in hemicellulose-derived compounds, and an elevated concentration of anhydro sugars such as levoglucosan. Overall, because SE leads to the irreversible loss of hemicellulose—a key precursor for liquid bio-oil—it adversely affects bio-oil quality and is therefore not favorable for bio-oil production.

## 5. Impacts of SE on Biomass Gasification

Biomass gasification technology is an efficient energy conversion method that transforms biomass feedstocks—such as agricultural and forestry residues, crop straw, and similar materials—into combustible gases [79,80]. In this process, biomass undergoes a series of thermochemical reactions, including pyrolysis, oxidation, reduction, and reforming, under high-temperature conditions, ultimately yielding syngas mainly composed of CO, H_2_, and CH_4_ [81,82]. Compared to coal, biomass represented by lignocellulose possesses several inherent characteristics: high oxygen content (40–50%), low energy density (15–20 kJ/kg), high content of alkali and alkaline earth metals (AAEMs), and challenges in collection, transportation, and storage, which limit its conversion for biomass energy [83,84]. Furthermore, lignocellulose is a complex composite consisting of various polymeric organic compounds. It contains a substantial amount of highly crystalline cellulose, and there exist multiple interaction forces between cellulose, hemicellulose, and lignin, rendering the lignocellulosic structure robust and difficult to convert. These characteristics can lead to undesirable effects during gasification: increased energy consumption, reduced gasification efficiency, lower heating value of syngas, higher tar content, as well as tendencies for pipeline clogging and corrosion of gasification equipment [85,86]. Consequently, researchers and industry professionals have increasingly shifted their focus toward novel gasification technologies such as biomass co-gasification [87,88] and modified gasification methods [89].

### 5.1. The Gasification Characteristics of Biomass Treated by SE

Biomass is mainly composed of lignin, hemicellulose, cellulose, extractives, and ash. The lignin content typically ranges from 10% to 20%, hemicellulose from 20% to 30%, and cellulose from 40% to 60% [90]. When gasified individually, the three major components exhibit distinct gasification characteristics. Lignin yields the highest gas production rate, with the resulting syngas containing relatively higher concentrations of H_2_ and CO_2_. Hemicellulose follows with the second-highest gas yield, producing syngas richer in CO and CH_4_. Cellulose has the lowest gas yield, and the composition of its syngas lies between that of hemicellulose and lignin [90,91]. However, in actual biomass, hemicellulose, cellulose, and lignin are interlinked through various chemical bonds and interactions, forming a complex structure [92,93]. Therefore, the gaseous composition of syngas from biomass gasification cannot be simply estimated based on the proportional content of these three components.

Gunarathne [94] studied the differences in high-temperature gasification between treated biomass and untreated biomass. Due to high fixed carbon and low volatile content, treated biomass undergo gasification dominated by the strongly endothermic boudouard reaction (C + CO_2_ → 2CO), resulting in a significantly temperature drop across the bed and yielding syngas with high CO, high hydrocarbons (e.g., CH_4_), and a high CO/CO_2_ ratio (~3.3), which collectively contribute to a HHV of the syngas [87,95]. In contrast, untreated biomass, characterized by higher volatile matter and lower carbon content, exhibits more uniform bed temperatures during gasification, where the water-gas shift reaction (CO + H_2_O → CO_2_ + H_2_) plays a more active role. Consequently, their syngas produced by treated biomass gasification is marked by high H_2_ content and a lower CO/CO_2_ ratio. Although both treated and untreated biomass achieve comparable cold-gas efficiencies (74–77%), treated biomass delivers a higher syngas energy yield. Furthermore, tar from treated biomass gasification contains a greater proportion of phenolic compounds, whereas tar derived from untreated biomass is predominantly composed of polycyclic aromatic hydrocarbons (PAHs). These distinct characteristics indicate that treated biomass is better suited for producing high-heating-value syngas, while untreated biomass is more favorable for generating hydrogen-rich syngas [96].

Dang [40] investigates the gasification behavior of biochar produced via a novel steam explosion (NSE) process. The gasification proceeds are divided into two distinct stages: devolatilization stage (25–600 °C) and gasification stage (>600 °C). High temperature (from 300 to 360 °C) and high pressure (from 2.5 to 3.5 MPa) enhance the biochar’s carbonization degree and thermal stability, raise the gasification onset temperature, and reduce its reactivity, with temperature exerting a more pronounced effect than pressure. Among these treated biomasses, R_2.5_-360 exhibits the most promising properties, including a fixed carbon content of 30.66%, high calorific value, and a low reaction energy barrier. This treated biomass exhibits significantly enhanced reactivity compared to untreated biomass, making it an effective substitute for pulverized coal and offering a viable pathway for the large-scale gasification of biomass.

### 5.2. The Influence of SE Pretreatment on Biomass Ash Properties

The ash content of biomass (typically 1–18%) is significantly lower than that of coal (5–48%), but the chemical composition of biomass ash is generally more complex [97,98]. Common trace elements found in biomass ash include Ca, K, Si, Mg, Al, Fe, P, Na, S, Mn, Ti, etc [99]. Moreover, the inorganic composition varies considerably across different types of biomass. Elements such as K, Ca, Na, Mg are classified as AAEMs. During gasification, these components exhibit a certain catalytic effect, aiding in the promotion of reactions [100]. Nevertheless, when present in high concentrations, their adverse impacts cannot be overlooked. Under high-temperature gasification or combustion conditions (800–1000 °C), AAEMs tend to volatilize and subsequently condense on the inner walls and piping of equipment, leading to corrosion issues [101]. Additionally, they can form low-melting-point compounds (often below 700 °C) with other ash constituents, significantly changing the melting and flow characteristics of the ash slag [102]. This affects the slag discharge process of gasification systems and may even result in operational failures in severe cases. Therefore, in the conversion of biomass energy, it is essential to fully consider the dual effects of its ash chemistry [97,103].

SE pretreatment tends to enrich inorganic elements in biomass, leading to an increase in ash content. Singhal [31] investigated the effects of different pretreatment methods on the composition and content of biomass ash. They found that SE pretreatment alone raised the ash content of biomass and resulted in the enrichment of inorganic elements in biomass treated by SE (14–36% for K, 8–53% for Na, 16–41% for S, 0–13% for Cl, 29–32% for Ca, 27–34% for Mg, 22–30% for Si, 24–39% for P, 6–58% for Fe and 0–10% for N). The enrichment of Na and K further contributed to a decrease in the ash fusion temperature of the biomass. On the other hand, SE disrupts the fibrous structure of biomass, making internal inorganic components more prone to volatilization, which can accelerate corrosion in gasification equipment [104]. In co-gasification processes, ash rich in Na and K tends to form O_3_-type oxides, while ash rich in Fe, Ca, and Mg tends to form P_2_-type oxides [105]. These two oxide types exhibit strong and weak catalytic properties, respectively. Therefore, compared to untreated biomass, steam-exploded pellets—containing higher content of AAEMs—may exert a stronger catalytic effect in co-gasification and are better suited for dry-ash gasifier.

When combined with water leaching, SE pretreatment can effectively remove inorganic elements from biomass and reduce its ash content. Singhal [31] integrated SE with water leaching to remove ash from biomass. Their study found that water leaching alone can dissolve surface ash, lowering the overall ash content of the biomass. Subsequent mechanical compression after leaching further enhanced ash removal efficiency. The combined processes of water leaching followed by SE (leaching–explosion) and SE followed by leaching (explosion–leaching) significantly improved ash removal efficiency (82–98% for K, 93–99% for Na, 80–99% for Cl, 34–90% for S, 3–77% for Mg, 43–63% for ash, 26–67% for P, and 19–63% for Ca) and increased the ash fusion temperature by 127–247 °C. Compared to other combined pretreatment methods, such as hydrothermal carbonization [106] combined with water leaching or torrefaction combined with leaching [20], the SE combined with water leaching approach achieved 30–42% higher ash removal efficiency. The sequence of SE and water leaching also influenced the ash removal efficiency: the leaching–explosion sequence showed slightly higher ash removal efficiency than the explosion–leaching sequence. This is largely attributed to the fact that water leaching induces swelling of biomass tissue cells, facilitating steam penetration during subsequent explosion and reducing resistance to the explosion process. Therefore, the production treated by the leaching–SE process exhibits higher ash fusion temperatures and lower ash content, making it more suitable for slag-tap gasifier.

In conclusion, SE represents an effective technique for biomass modification, capable of adjusting ash content to a certain degree and holding considerable promise for biomass energy applications.

## 6. Research Reequipment and Future Perspectives

The large-scale commercialization of the SE process continues to face several key technical problems. To advance toward viable industrial implementation, future research and process development should critically address the following problems.

1. **Reaction kinetics of biomass in the steam cooking stage**. The steam cooking stage, as a critical step in the SE process, directly determines the characteristics of biomass fuel. High-Pressure Steam Thermogravimetric Analysis (HP-Steam TGA) is an advanced in situ characterization technique that integrates conventional TGA with a high-pressure steam reaction environment. It can quantitatively reveal the degradation kinetics, interaction mechanisms, and reaction pathways of the main biomass components (hemicellulose, cellulose, and lignin) during the steam cooking stage. By precisely controlling key parameters such as steam pressure, temperature, and time, HP-Steam TGA enables real-time and continuous monitoring of mass loss in biomass under high-pressure steam. This provides an essential theoretical basis for establishing accurate kinetic models of the SE reaction and optimizing the process parameters.
2. **Research on SH-SE**. Traditional SE technology typically uses saturated steam as the medium for SE pretreatment. However, saturated steam has a high heat transfer coefficient and significant moisture content, which tends to cause latent heat loss during pipeline transportation and aggravates equipment corrosion, leading to energy waste and higher maintenance costs. In contrast, superheated steam has a heat transfer coefficient only 1/20 that of saturated steam and is less prone to condensation during transport, which can notably mitigate pipeline corrosion. This characteristic makes it particularly suitable for the long-distance transportation of industrial waste heat to SE units, thereby enabling efficient utilization of waste heat and reducing maintenance costs for the pipeline system.
3. **Research on heat and mass transfer enhancement in SE pretreatment**. In traditional batch reactors of SE, the presence of air within the cooking chamber is unavoidable. As a non-condensable gas, air accumulates at the gas–solid interface between the steam and the biomass, forming an insulating gas film. This film not only introduces additional thermal resistance to heat transfer but also impedes moisture migration, significantly reducing the heat and mass transfer capacity of steam. Therefore, to address the issue of uneven heating caused by the buildup of non-condensable gases in large-scale SE systems, there is an urgent need to develop novel technological approaches to reduce the concentration of these gases and enhance the heat and mass transfer capabilities of the process.

## 7. Discussion

SE is widely recognized as an efficient, environmentally sustainable, and cost-effective pretreatment technology for lignocellulosic biomass, offering considerable advantages in both biorefining and energy conversion applications. The process entails subjecting biomass to saturated steam at enhanced temperatures and pressures (typically 160–260 °C), followed by swift decompression that induces mechanical fiber disruption. This treatment effectively dismantles the rigid lignocellulosic matrix, thereby enhancing cellulose accessibility and enzymatic digestibility. In energy applications, SE-pretreated biomass can be densified into “black pellets,” which demonstrate superior tensile strength, durability, and water resistance, thereby assisting long-distance transportation and outdoor storage. Compared to conventional “white pellets,” black pellets possess higher energy density and can be co-milled and co-fired with coal in existing power plant boilers without necessitating retrofitting. In addition, combustion of these pellets results in reduced emissions of particulate matter, NOx, and SO_2_ relative to coal alone. SE pretreatment has also been shown to improve the quality of biochar, bio-oil, and syngas in subsequent pyrolysis and gasification processes. Also, anaerobic digestion of SE-pretreated biomass can enhance methane yields by 8.6% to 19.6%. This technology is applicable to a broad range of feedstocks—including agricultural residues, forestry waste, and industrial byproducts—and features relatively low energy consumption, with a maximum reported input of 1.4 kJ/g. Due to the improved fuel characteristics imparted by pretreatment, steam-exploded particles exhibit excellent performance in pyrolysis and gasification, with increased specific surface and enhanced reactivity, significantly improving thermal conversion efficiency and thereby optimizing the conversion of biomass energy.

From an engineering standpoint, scaling up SE technology remains challenging owing to the complexity of reactor design and the difficulties associated with controlling heat and mass transfer, and residence time distribution during the transition from laboratory to industrial scales. The physicochemical heterogeneity of biomass feedstocks further complicates process optimization, as optimal operating parameters must be empirically determined for each material, limiting the technology’s general applicability. Economically, standalone production of fuel ethanol from SE-pretreated biomass is often marginally profitable; thus, improving overall process economics depends on the co-production of high-value byproducts such as xylooligosaccharides and phenolic compounds. Future research directions include integrating downstream post-treatment steps—such as leaching and washing—to mitigate inhibitory compound effects, developing novel reactor configurations (e.g., SHSE and continuous screw reactors), and further optimizing energy efficiency and economic viability for commercial-scale implementation.

## Figures and Tables

**Table 4:** The description of CTR in Figure 5 [58]. Copyright 2026 American Chemical Society.

ID	Device	Description
1	extruder motor	11 rpm (fixed), 10 cm pitch and 4 flights screw
2	hopper	LB feeding
3	extruder body	2:1 reduction ratio
4	conveyor motor	9.2 rpm max, 10 cm pitch and 14 flights screw
5	CTR body	1.6 m long, 10 cm diameter
6	saturated steam input	180 °C, 1.034 MPa
7	discharge valves	explosion every 1 min
8	discharge box	receives exploded LB

## Data Availability

No new data were created or analyzed in this study. Data sharing is not applicable to this article.

## Abbreviations

SE	steam explosion
VOCs	volatile organic compounds
HHV	higher heating value
EFB	empty fruit bunch
PKS	palm kernel shell
DF	douglas fir
ICSE	instant catapult steam explosion
EPD	explosion power density
POP	pilot-operated pneumatic
SCR	screw conveyor reactor
TGA	thermogravimetric analysis
PAHs	polycyclic aromatic hydrocarbons
NSE	novel steam explosion
TG	thermogravimetry
DTG	derivative thermogravimetry
AAEMs	alkali and alkaline earth metals
HP-Steam TGA	high-pressure steam thermogravimetric analysis
SH-SE	superheated steam explosion pretreatment

**Notations**

***R*** **_0_**	severity factor [min]
***t***	the time of steam explosion [min]
***T***	the temperature of reaction [°C]
$m^{\prime }$	the mass of steam consumed by per unit mass of dry materials [kg kg^−1^]
***Q_t_***	the total energy consumption of the steam explosion process [kJ]
***h_ge_***	enthalpy of steam at final stage [kJ kg^−1^]
***h_f0_***	enthalpy of water at initial stage [kJ kg^−1^]
***m***	amount of material [kg]

## References

[1] Auxenfans T., Crônier D., Chabbert B., Paës G. (2017). Understanding the structural and chemical changes of plant biomass following steam explosion pretreatment. Biotechnol. Biofuels.

[2] Pang S. (2019). Advances in thermochemical conversion of woody biomass to energy, fuels and chemicals. Biotechnol. Adv..

[3] Ibitoye S.E., Mahamood R.M., Jen T.-C., Loha C., Akinlabi E.T. (2023). An overview of biomass solid fuels: Biomass sources, processing methods, and morphological and microstructural properties. J. Bioresour. Bioprod..

[4] Meng X., Xu X., Hu S., Chu R., Li X., Li W., Yu S., Sun Y. (2025). Changes in the pore structure and oxygen-containing functional groups of lignite under steam explosion and their influence on water molecule resorption. Colloids Surf. A Physicochem. Eng. Asp..

[5] Sharma G., Kumar M., Pandey A.K., Kumari S., Singh A., Ansari N.A., Gaur N.A. (2025). Pretreatment technologies for valorisation of lignocellulosic biomass towards sustainable biorefineries: Comprehensive insights and advances. Bioresour. Technol. Rep..

[6] Yu Y., Wu J., Ren X., Lau A., Rezaei H., Takada M., Bi X., Sokhansanj S. (2022). Steam explosion of lignocellulosic biomass for multiple advanced bioenergy processes: A review. Renew. Sustain. Energy Rev..

[7] Chen H., Liu J., Chang X., Chen D., Xue Y., Liu P., Lin H., Han S. (2017). A review on the pretreatment of lignocellulose for high-value chemicals. Fuel Process. Technol..

[8] Paletta R., Candamano S., Agblevor F.A., Castro Y.A. (2026). Effect of mild alkaline hydrogen peroxide pretreatment on the biomethanation kinetics of *Sargassum* spp. biomass. Biomass Bioenergy.

[9] Novia N., Jannah A.M., Melwita E., Fudholi A., Pareek V.K. (2026). Advances and challenges in deep eutectic solvents pretreatment technologies for bioethanol production from lignocellulosic biomass: A comprehensive review. Renew. Sustain. Energy Rev..

[10] Sun Y., Cheng J. (2002). Hydrolysis of lignocellulosic materials for ethanol production: A review. Bioresour. Technol..

[11] Mosier N., Wyman C., Dale B., Elander R., Lee Y.Y., Holtzapple M., Ladisch M. (2005). Features of promising technologies for pretreatment of lignocellulosic biomass. Bioresour. Technol..

[12] Brethauer S., Studer M.H. (2015). Biochemical Conversion Processes of Lignocellulosic Biomass to Fuels and Chemicals—A Review. Chimia.

[13] Patel M., Zhang X., Kumar A. (2016). Techno-economic and life cycle assessment on lignocellulosic biomass thermochemical conversion technologies: A review. Renew. Sustain. Energy Rev..

[14] Chang P., Wang J., Chen Z., Dong J., Ding R., Yang T., Zhang J. (2025). Synthesis of porous carbon induced by hydrothermal crosslinking reaction from fulvic acid for organic supercapacitor with high energy density by improving the voltage window. Chem. Eng. J..

[15] Zabed H.M., Akter S., Yun J., Zhang G., Awad F.N., Qi X., Sahu J.N. (2019). Recent advances in biological pretreatment of microalgae and lignocellulosic biomass for biofuel production. Renew. Sustain. Energy Rev..

[16] Qian L., Wang Y., Ni J., Wang S., Gu H. (2023). Research progress of modification method and application of biomass hydrochar. J. China Coal Soc..

[17] Khandelwal K., Seraj S., Nanda S., Azargohar R., Dalai A.K. (2024). Subcritical water conversion of biomass to biofuels, chemicals and materials: A review. Environ. Chem. Lett..

[18] Jacquet N., Maniet G., Vanderghem C., Delvigne F., Richel A. (2015). Application of Steam Explosion as Pretreatment on Lignocellulosic Material: A Review. Ind. Eng. Chem. Res..

[19] Sarker T.R., Pattnaik F., Nanda S., Dalai A.K., Meda V., Naik S. (2021). Hydrothermal pretreatment technologies for lignocellulosic biomass: A review of steam explosion and subcritical water hydrolysis. Chemosphere.

[20] Abelha P., Mourão Vilela C., Nanou P., Carbo M., Janssen A., Leiser S. (2019). Combustion improvements of upgraded biomass by washing and torrefaction. Fuel.

[21] Pei X., Xie J., Fan M. (2026). NaOH-ethanol pretreatment for cellulosic ethanol: A techno-economic comparison with Dilute acid and Steam explosion processes. Biomass Bioenergy.

[22] Chornet E., Overend R.P., Ruiz H.A., Thomsen M.H., Trajano H.L. (2017). How the Severity Factor in Biomass Hydrolysis Came About. Hydrothermal Processing in Biorefineries: Production of Bioethanol and High Added-Value Compounds of Second and Third Generation Biomass.

[23] Cai C., Wang L., Wang G., Hao J., Bai X., Wang Z., Wang D. (2020). Effects of dry explosion pretreatment on physicochemical and fuel properties of hybrid pennisetum (*Pennisetum americanum* × *P. purpureum*). Bioresour. Technol..

[24] Zhang Y., Chen H. (2012). Multiscale modeling of biomass pretreatment for optimization of steam explosion conditions. Chem. Eng. Sci..

[25] Heidari M., Salaudeen S., Arku P., Acharya B., Tasnim S., Dutta A. (2021). Development of a mathematical model for hydrothermal carbonization of biomass: Comparison of experimental measurements with model predictions. Energy.

[26] Sui W., Chen H. (2014). Multi-stage energy analysis of steam explosion process. Chem. Eng. Sci..

[27] Sui W., Chen H. (2016). Effects of water states on steam explosion of lignocellulosic biomass. Bioresour. Technol..

[28] Barciela P., Perez-Vazquez A., Carpena M., Seyyedi-Mansour S., Donn P., Fraga-Corral M., Otero P., Xiao J., Simal-Gandara J., Prieto M.A. (2023). Insight into Steam Explosion Pretreatment of Sugarcane Bagasse for Bioethanol Production. Eng. Proc..

[29] Lancha J.P., Perré P., Colin J., Lv P., Ruscassier N., Almeida G. (2021). Multiscale investigation on the chemical and anatomical changes of lignocellulosic biomass for different severities of hydrothermal treatment. Sci. Rep..

[30] Wang S., Ru B., Lin H., Sun W. (2015). Pyrolysis behaviors of four O-acetyl-preserved hemicelluloses isolated from hardwoods and softwoods. Fuel.

[31] Singhal A., Roslander C., Goel A., Ismailov A., Erdei B., Wallberg O., Konttinen J., Joronen T. (2024). Combined leaching and steam explosion pretreatment of lignocellulosic biomass for high quality feedstock for thermochemical applications. Chem. Eng. J..

[32] Ruiz H.A., Conrad M., Sun S.-N., Sanchez A., Rocha G.J.M., Romaní A., Castro E., Torres A., Rodríguez-Jasso R.M., Andrade L.P. (2020). Engineering aspects of hydrothermal pretreatment: From batch to continuous operation, scale-up and pilot reactor under biorefinery concept. Bioresour. Technol..

[33] Biswas A.K., Umeki K., Yang W., Blasiak W. (2011). Change of pyrolysis characteristics and structure of woody biomass due to steam explosion pretreatment. Fuel Process. Technol..

[34] Biswas A.K., Yang W., Blasiak W. (2011). Steam pretreatment of Salix to upgrade biomass fuel for wood pellet production. Fuel Process. Technol..

[35] Lam P.S., Sokhansanj S., Bi X., Lim C.J., Melin S. (2011). Energy Input and Quality of Pellets Made from Steam-Exploded Douglas Fir (*Pseudotsuga menziesii*). Energy Fuels.

[36] Zandersons J., Gravitis J., Zhurinsh A., Kokorevics A., Kallavus U., Suzuki C.K. (2004). Carbon materials obtained from self-binding sugar cane bagasse and deciduous wood residues plastics. Biomass Bioenergy.

[37] Alhwayzee M.H., Imran A.M., Nassrullah K.S. (2020). Evaluation of Solid Biomass Fuel for Some Iraqi Agricultural Wastes Using Proximate and Ultimate Analyses. IOP Conf. Ser. Mater. Sci. Eng..

[38] Shaw M.D., Karunakaran C., Tabil L.G. (2009). Physicochemical characteristics of densified untreated and steam exploded poplar wood and wheat straw grinds. Biosyst. Eng..

[39] Borén E., Yazdanpanah F., Lindahl R., Schilling C., Chandra R.P., Ghiasi B., Tang Y., Sokhansanj S., Broström M., Larsson S.H. (2017). Off-Gassing of VOCs and Permanent Gases during Storage of Torrefied and Steam Exploded Wood. Energy Fuels.

[40] Wattan R., Zamiri M.A., Nikkhah Dafchahi M., Newkirk R.W., Acharya B., Dalai A.K. (2025). Biofuel development from flax straw: Torrefaction, steam explosion, and pelletization of treated biomass. Ind. Crops Prod..

[41] Dang H., Xu R., Zhang J., Wang M., Zhang J., Wang M., Wan K., Jia G., Lan D. (2026). Waste to energy: Gasification characteristics and conversion mechanism of biomass char in the novel steam explosion process for blast furnace injection. Int. J. Hydrog. Energy.

[42] He H., Wang Y., Sun Y., Sun W., Wu K. (2024). From raw material powder to solid fuel pellet: A state-of-the-art review of biomass densification. Biomass Bioenergy.

[43] Ige A.R., Łaska G. (2025). Production of antioxidant additives and biochar pellets from the Co-pyrolysis of agricultural biomass: A review. Renew. Sustain. Energy Rev..

[44] Shao S., Jin Z., Wen G., Iiyama K. (2009). Thermo characteristics of steam-exploded bamboo (*Phyllostachys pubescens*) lignin. Wood Sci. Technol..

[45] Mawusi S.K., Shrestha P., Xue C., Liu G. (2023). A comprehensive review of the production, adoption and sustained use of biomass pellets in Ghana. Heliyon.

[46] Lam P.S., Lam P.Y., Sokhansanj S., Lim C.J., Bi X.T., Stephen J.D., Pribowo A., Mabee W.E. (2015). Steam explosion of oil palm residues for the production of durable pellets. Appl. Energy.

[47] Onyenwoke C., Tabil L.G., Dumonceaux T., Mupondwa E., Cree D., Li X., Onu Olughu O. (2023). Technoeconomic Analysis of Torrefaction and Steam Explosion Pretreatment Prior to Pelletization of Selected Biomass. Energies.

[48] Chen H., Sui W., Ruiz H.A., Thomsen M.H., Trajano H.L. (2017). Steam Explosion as a Hydrothermal Pretreatment in the Biorefinery Concept. Hydrothermal Processing in Biorefineries: Production of Bioethanol and High Added-Value Compounds of Second and Third Generation Biomass.

[49] Yu Z. (2015). Experiment Studies on Instant Catapult Steam Explosion: Technology and Effect Mechanisms to Biomass Conversion. Ph.D. Thesis.

[50] Yu Z., Zhang B., Yu F., Xu G., Song A. (2012). A real explosion: The requirement of steam explosion pretreatment. Bioresour. Technol..

[51] Zhong Y., Wu Y., Zhu R., Lin Q., Wang X., Ren J., Wang H., Du F. (2025). Low energy consumption for instantaneous catapult steam explosion on the enzymatic hydrolysis of wheat straw. Ind. Crops Prod..

[52] Cai C., Wang G., Wang L., Zhang X. (2023). Rethinking the role of steam explosion on biomass composition, structure, and saccharification with new-designed pilot-operated pneumatic decompression mode. Biomass Convers. Biorefinery.

[53] Wang C., Lin M., Yang Q., Fu C., Guo Z. (2023). The Principle of Steam Explosion Technology and Its Application in Food Processing By-Products. Foods.

[54] Elander R.T. (2013). Experimental Pretreatment Systems from Laboratory to Pilot Scale. Aqueous Pretreatment of Plant Biomass for Biological and Chemical Conversion to Fuels and Chemicals.

[55] Ramirez-Cabrera P.A., Lozada-Castro J.J., Guerrero-Fajardo C.A. (2024). Screw reactor design for potato peel pretreatment using the steam explosion. Bioresour. Technol..

[56] Pielhop T., Amgarten J., von Rohr P.R., Studer M.H. (2016). Steam explosion pretreatment of softwood: The effect of the explosive decompression on enzymatic digestibility. Biotechnol. Biofuels.

[57] Pérez Pimienta J.A., Papa G., Rodriguez A., Barcelos C.A., Liang L., Stavila V., Sanchez A., Gladden J.M., Simmons B.A. (2019). Pilot-scale hydrothermal pretreatment and optimized saccharification enables bisabolene production from multiple feedstocks. Green Chem..

[58] Jaramillo I., Sanchez A. (2018). Mass Flow Dynamic Modeling and Residence Time Control of a Continuous Tubular Reactor for Biomass Pretreatment. ACS Sustain. Chem. Eng..

[59] Ma P., Lan J., Feng Y., Liu R., Qu J., He H. (2015). Effects of Continuous Steam Explosion on the Microstructure and Properties of Eucalyptus Fibers. BioResources.

[60] Zimbardi F., Viggiano D., Nanna F., Demichele M., Cuna D., Cardinale G., Davison B.H., Finkelstein M. (1999). Steam Explosion of Straw in Batch and Continuous Systems. Twentieth Symposium on Biotechnology for Fuels and Chemicals: Presented as Volumes 77–79 of Applied Biochemistry and Biotechnology Proceedings of the Twentieth Symposium on Biotechnology for Fuels and Chemicals Held, Gatlinburg, TN, USA;, 3–7 May 1998.

[61] Chen L., Wang H., Tu Z., Hu J., Wu F. (2024). Renewable fuel and value-added chemicals potential of reed straw waste (RSW) by pyrolysis: Kinetics, thermodynamics, products characterization, and biochar application for malachite green removal. Renew. Energy.

[62] Abioye K.J., Falua K.J., Rezaee M., Zamiri M.A., Zou F., Acharya B. (2025). Global insights into biomass pyrolysis mechanisms: A scientometric and mechanistic approach. Results Eng..

[63] Rathi N., Das T. (2025). Exploring biomass pyrolysis for sustainable hydrogen-rich gas production. Biomass Bioenergy.

[64] Kumar R., Strezov V., Weldekidan H., He J., Singh S., Kan T., Dastjerdi B. (2020). Lignocellulose biomass pyrolysis for bio-oil production: A review of biomass pre-treatment methods for production of drop-in fuels. Renew. Sustain. Energy Rev..

[65] Shen D.K., Gu S., Bridgwater A.V. (2010). Study on the pyrolytic behaviour of xylan-based hemicellulose using TG–FTIR and Py–GC–FTIR. J. Anal. Appl. Pyrolysis.

[66] Zhang X., Zhang Y., Zhang S., Yao L., Hao Y. (2025). Lignocellulosic biomass pyrolysis: A review on the pretreatment and catalysts. Fuel Process. Technol..

[67] Yang H., Yan R., Chen H., Lee D.H., Zheng C. (2007). Characteristics of hemicellulose, cellulose and lignin pyrolysis. Fuel.

[68] Chen L., Liao Y., Guo Z., Cao Y., Ma X. (2019). Products distribution and generation pathway of cellulose pyrolysis. J. Clean. Prod..

[69] Wang S., Dai G., Yang H., Luo Z. (2017). Lignocellulosic biomass pyrolysis mechanism: A state-of-the-art review. Prog. Energy Combust. Sci..

[70] Zhang J., Cao R., You M., Liu Y., Han K. (2026). Pyrolysis characteristics and kinetics of typical Chinese herbal medicine residues. Biomass Bioenergy.

[71] Gu X., Liu C., Jiang X., Ma X., Li L., Cheng K., Li Z. (2014). Thermal behavior and kinetics of the pyrolysis of the raw/steam exploded poplar wood sawdust. J. Anal. Appl. Pyrolysis.

[72] Tianbao R., Xiaoqin M., Guizhuan X., Andong S., Bailiang Z. (2011). Pyrolysis characteristics and kinetic ananlysis of corn stalks by steam explosion. Trans. Chin. Soc. Agric. Eng..

[73] Mohan D., Pittman C.U., Steele P.H. (2006). Pyrolysis of Wood/Biomass for Bio-oil:  A Critical Review. Energy Fuels.

[74] Guida M.Y., Hannioui A. (2016). A review on thermochemical treatment of biomass: Pyrolysis of olive mill wastes in comparison with other types of biomass. Prog. Agric. Eng. Sci..

[75] Charisteidis I., Lazaridis P., Fotopoulos A., Pachatouridou E., Matsakas L., Rova U., Christakopoulos P., Triantafyllidis K. (2019). Catalytic Fast Pyrolysis of Lignin Isolated by Hybrid Organosolv—Steam Explosion Pretreatment of Hardwood and Softwood Biomass for the Production of Phenolics and Aromatics. Catalysts.

[76] Sivriu A.-M., Jinescu G., Sapunaru O., Tirpan Cioroiu D.-R., Koncsag C. (2019). Pyrolysis of Waste Palm Oil in the Presence of Steam. Rev. Chim.-Buchar.-Orig. Ed..

[77] Wang H., Srinivasan R., Yu F., Steele P., Li Q., Mitchell B. (2011). Effect of Acid, Alkali, and Steam Explosion Pretreatments on Characteristics of Bio-Oil Produced from Pinewood. Energy Fuels.

[78] Wang H., Srinivasan R., Yu F., Steele P., Li Q., Mitchell B., Samala A. (2012). Effect of Acid, Steam Explosion, and Size Reduction Pretreatments on Bio-oil Production from Sweetgum, Switchgrass, and Corn Stover. Appl. Biochem. Biotechnol..

[79] Zhang Y., Hou D., Sun X., Zhu X., Yan B., Chen G. (2025). Different pretreatment of biomass for gasification: A critical review. J. Energy Inst..

[80] Joshua Abioye K., Rajamanickam R., Ogunjinmi T., Paul S., Selvasembian R., Ighalo J.O. (2025). Advancements in biomass waste conversion to sustainable biofuels via gasification. Chem. Eng. J..

[81] Alsaeedi S., Zhang R., Yan B., Wang Z., Li J., Al-Hakeem B., Chen G. (2026). A review of biomass energy and gasification: Bibliometrics, hotspots and emerging trends. Biomass Bioenergy.

[82] Mustapha S.I., Anekwe I.M.S., Akpasi S.O., Muritala K.B., Tetteh E.K., Joel A.S., Isa Y.M. (2025). Biomass conversion for sustainable hydrogen generation: A comprehensive review. Fuel Process. Technol..

[83] Lolja S.A., Haxhi H., Dhimitri R., Drushku S., Malja A. (2002). Correlation between ash fusion temperatures and chemical composition in Albanian coal ashes. Fuel.

[84] Vassilev S.V., Baxter D., Vassileva C.G. (2013). An overview of the behaviour of biomass during combustion: Part I. Phase-mineral transformations of organic and inorganic matter. Fuel.

[85] Wei J., Zhang J., Zhang Z., Gao N., Zhang Y., Cui D., Lyu P., Song X., Yu G. (2025). Research progress on catalytic behavior of biomass ash during gasification of carbonaceous feedstocks. Clean Coal Technol..

[86] Zhan T. (2021). Experiment Study on Typical Biomass Pyrolysis and Gasification. Master’s Thesis.

[87] Qi J., Yao J., Chen G., Yi W., Yan B., Cheng Z., Yao Y., Liu J., Liu X., Bi C. (2023). Investigation progress on the synergy between coal and biomass during co-gasification. J. Fuel Chem. Technol..

[88] Liang Y., Yalkunjan T., Gao G., Zhong M., Dai Z., Li J., Liu Y., Zhang X., Yuan W., Xu X. (2025). Migration and transformation of alkali and alkaline earth metals during co-gasification of biomass and coal. J. Fuel Chem. Technol..

[89] Attasophonwattana P., Sitthichirachat P., Siripaiboon C., Ketwong T., Khaobang C., Panichnumsin P., Ding L., Areeprasert C. (2022). Evolving circular economy in a palm oil factory: Integration of pilot-scale hydrothermal carbonization, gasification, and anaerobic digestion for valorization of empty fruit bunch. Appl. Energy.

[90] Tian T. (2017). Effect of Composition on HydrogenProduction of Biomass Gasification. Master’s Thesis.

[91] Suparmin P., Purwanti N., Oscar Nelwan L., Tambunan A.H. (2024). Syngas production by biomass gasification: A meta-analysis. Renew. Sustain. Energy Rev..

[92] Xu R., Doskaliuk N., Pang B., Xu J., Xu W., Xu C., Antonietti M., Filonenko S. (2025). Hemicellulose from mild extraction of biomass: Revealing structural insights and advancing potential value. Carbohydr. Polym. Technol. Appl..

[93] Li L., Wang J., Chen Z., Dong J., Chang P., Zhang J., Yang T., Ding R. (2025). Preparation of sodium lignosulfonate-based porous carbon for supercapacitors with outstanding rate capacity and high voltage. Chem. Eng. J..

[94] Gunarathne D.S., Mueller A., Fleck S., Kolb T., Chmielewski J.K., Yang W., Blasiak W. (2014). Gasification characteristics of steam exploded biomass in an updraft pilot scale gasifier. Energy.

[95] Pitkäoja A., Ritvanen J., Winter F. (2025). Model-based investigation of biomass CO_2_ gasification to biomass-to-X plant utilising dual fluidised bed gasification. Chem. Eng. J..

[96] Zahra A.C.A., Alahakoon A.M.Y.W., Zhu L., Prakoso T., Abudula A., Guan G. (2025). Biochar-assisted gasification of raw biomass: A review on the reactivity and synergistic effect on tar reforming. Resour. Chem. Mater..

[97] Abioye K.J., Harun N.Y., Sufian S., Yusuf M., Jagaba A.H., Ekeoma B.C., Kamyab H., Sikiru S., Waqas S., Ibrahim H. (2024). A review of biomass ash related problems: Mechanism, solution, and outlook. J. Energy Inst..

[98] Munawar M.A., Khoja A.H., Naqvi S.R., Mehran M.T., Hassan M., Liaquat R., Dawood U.F. (2021). Challenges and opportunities in biomass ash management and its utilization in novel applications. Renew. Sustain. Energy Rev..

[99] Zhou T., Zhang W., Shen Y., Luo S., Ren D. (2023). Progress in the change of ash melting behavior and slagging characteristics of co-gasification of biomass and coal: A review. J. Energy Inst..

[100] Zou X., Wang P., Cai M., Guo L., Zhang Y., Xie G., Zhai M. (2026). Mechanistic study of AAEM-catalyzed steam gasification of oxygen-containing functional groups in biomass char based on DFT. J. Anal. Appl. Pyrolysis.

[101] Hupa M., Karlström O., Vainio E. (2017). Biomass combustion technology development—It is all about chemical details. Proc. Combust. Inst..

[102] Li X., Zhang R., Yao Q., Meng X., Sun Z., Li Z., Qiu F., Li P., Zhou E. (2025). Catalytic effects of key compositions in biomass ashes on coal gasification reactivity and structural evolution characteristic during gasification process. J. Clean. Prod..

[103] Yao X., Zhao Z., Chen S., Zhou H., Xu K. (2020). Migration and transformation behaviours of ash residues from a typical fixed-bed gasification station for biomass syngas production in China. Energy.

[104] Chen L., Xu Z., Xie X., Shen Z., Xu J., Dai Z., Liu H. (2026). Regulation and migration behaviour of chlorine during corn straw gasification. Fuel.

[105] Xiao H., Wang Y., Cai Z., Zhang J., Yu G. (2024). The synergistic effecting mechanisms of biomass pyrolysis, biomass char gasification, and biomass ash on CO_2_ co-gasification of biomass and high-sulfur petroleum coke. Fuel.

[106] Hansen L.J., Fendt S., Spliethoff H. (2022). Impact of hydrothermal carbonization on combustion properties of residual biomass. Biomass Convers. Biorefinery.

